# Life Satisfaction and mobility: Their associations with career attitudes, and health-related factors among postgraduates having studied in universities intra EU and outside EU

**DOI:** 10.1186/s12889-019-7913-8

**Published:** 2020-01-06

**Authors:** Angela Odero, Louis Chauvel, Anne Hartung, Etienne le Bihan, Michèle Baumann

**Affiliations:** Institute for Research on Socio-Economic Inequalities – axis Health inequalities. Research unit INSIDE, University of Luxembourg, Belval Campus, L-4366 Esch-sur-Alzette, Luxembourg

**Keywords:** Life satisfaction, Student mobility abroad, Postgraduates, Health satisfaction, Career attitudes, Cultural capital

## Abstract

**Background:**

University postgraduates’ mobility towards, and outside the EU is continuously increasing, creating a competitive context in which maintaining a high life satisfaction (LS) is a public health challenge. However, the relationship between LS and its determinants among this population are under-documented. Our aims were to measure LS indicators of mobile postgraduates (Intra EU: Who pursue part of their studies in Europe; Outside EU: Who study outside of Europe) versus non-mobile (pursue their studies in Luxembourg), and to analyze the associations between LS and career attitudes, socioeconomic characteristics, and health-related factors for each group.

**Method:**

Six hundred and sixty-four (644) students obtained financial aid from the Luxembourgish government independent of their family’s socioeconomic situation. Contacted by post, they completed an online questionnaire. Analyses included a multiple linear regression model in which only significant relationships (*p* < 0.05) were used.

**Results:**

Three groups were created: Mobile intra EU (*n* = 381), mobile outside EU (*n* = 43) and non-mobile (*n* = 66) postgraduates. Health satisfaction was positively linked to LS, in all groups. Among the mobile outside EU group, majority (63.2%) were men and 57.9% did not live alone - health was the only determinant which contributed to their LS. Among the mobile intra EU, majority (57.8%) were women, and 64.3% not living alone. Autonomy and career adaptability attitudes were positively associated with their LS (b: 0.210 and 0.119, respectively), whereas the worry factor was negatively (b: − 0.153 and -0.159) associated. The non-mobile, were the oldest of the three groups. Majority (51.6%) were women, and 93.7% did not live alone. Career optimism and planning attitudes were positively correlated to their LS (regression parameter estimates (b: 0.400 and 0.212, respectively).

**Conclusions:**

Attention should be devoted to the LS of local and cosmopolitan students, as it seems to be a relevant health indicator. Overall, the farther the mobility was, the higher the postgraduates’ general LS (8.5/10) was; this indicator was higher than the LS indicator for the age group 25–34 years 7.53/10 (EU-28, in 2013). University’ services could promote the development of career projects and the promotion of health to enhance postgraduates’ LS. University policy makers need to ensure this for all students.

## Background

An increasing number of international students profit from the ease of mobility within and outside the EU. University mobility experience is a time to capitalize on personal abilities; hence students are likely to be driven by strategies linked to employability or by the prospect of an enriching experience abroad. However, mobility can also pose a risk to physical and mental functioning [1], and life satisfaction (LS) [2]. For instance, the experience of mobility abroad may produce protective (e.g. positive affect, optimism, happiness), or detrimental effects (e.g. anxiety, hostility, anger) [3]. This raises the question whether the level of LS, which represents the perceived degree of discrepancy between individual aspirations and achievements, and contentment [4, 5], differs between mobile intra EU and mobile outside EU. [Intra EU: Students who pursue part of their studies (example, Erasmus semester) in a European country, different from their home country; Outside EU: Students who pursue part of their studies (example, Erasmus global exchange) outside Europe].

Leaving home to live in another country and adjusting to a new social and cultural environment can increase the prevalence of social isolation, depression, and anxiety coupled with hazardous behaviours, such as smoking, alcohol consumption, loss of appetite, fatigue, and sleep difficulties [6, 7]. These risk factors are related to suicidal and nonfatal suicidal behaviors among university students [8]. Acculturative stressors and pressures [9] have negative consequences in terms of reduced mental health and students’ LS [10]. Life dissatisfaction has a long-term effect on the risk of suicide, which seems to be partly mediated by poor health behaviours [11]. Social and familial pressures push postgraduates to join mobility aboard because they expect the outcome to be beneficial to their future careers, such as building an international social network. In the health field, an increased interest in an active social network (also known as social capital) has been explored, and suggests that social capital is positively related to health [12].

The expansion of knowledge about other cultures in order to gain competitive advantage in the international labor market has propelled young people to study not only in other European countries, but also in other regions of the world [13]. The experiences of mobility abroad are likely to enhance students’ career prospects in terms of securing higher job interview rates, job offers and accelerating career progression [14, 15]. The employability of young graduates is increasingly dependent upon their ability to maintain ‘positional advantage’ and to acquire skills and behaviours appreciated in a labour market [16], for instance, autonomy and adaptability. These acquired competences can be explained in terms of social and cultural capitals, whose potential effects on health have been studied [12, 17]. For instance, three levels of cultural capital that could offer a conceptual framework through which to explain student mobility have been identified: Institutionalized (educational credentials, specialized knowledge, etc.), embodied (personality, skills, etc.), and objectified (clothes, other belongings, etc.) [17]. Other studies emphasize a negative value of foreign cultural capital, stressing that local cultural capital may be more functionally relevant [18, 19]. Similarly, reports about students’ LS, before and after their mobility, and the effects of mobility on LS have also been mixed, with some suggesting a positive effect, others a negative effect, and others still, no effect at all [20]. Factors such as cultural empathy, social initiative, intercultural competences, language ability, knowledge about the host country, number of friends, satisfaction with finances, all of which intervene on LS, could explain these differences [21, 22].

Students who decide to study abroad differ from those who undertake their studies in their home country [23]. Embodied cultural capitals such as career attitudes may be represented by an individual’s predisposition to employ competences and knowledge related to employability, acquired during their education. Students with a higher dynamic career attitude are more likely to identify future job opportunities more easily, by adapting their competences to the post-university transition [24]. Students may feel more comfortable managing and controlling important aspects of their lives and are more likely to be satisfied with their achievements [25].

International students may experience cultural shock and academic cultural shock. The gap between cosmopolitan versus local types is documented in the literature, with ‘locals’ described as disposing of stronger local interconnections or social capital, whereas ‘cosmopolitans’ are obliged to compete in an open competitive society, where they have to develop their own mobile capital (degree, skills, and resources). It can be argued that the former prefer the protection of communities (family, neighbourhood, etc.) while the latter search for stimulation and personal development in demanding contexts, potentially risky for health and cognitive resources [26–28]. However, in this competitive context, the exploration of LS for the local/non-mobile and for the mobile/cosmopolitan postgraduates and its determinants has been little investigated.

Between 1971 and 2011, the number of higher education students in the world increased five-fold, an upward trend that persists to date. Between 2000 and 2011, the number of foreign students enrolled in tertiary education doubled. The same trend is mirrored in Organization for Economic Co-operation and Development (OECD) countries as well. As a result, over the past two decades, the profile of tertiary education students and graduates has included more international students, and the preferred fields of study have changed. Accordingly, universities are introducing new programs as they are challenged to adapt their programs not only to the global economy, but also to the interests and priorities of the growing and diverse student population [29]. This may reflect the competitive nature of the labor market. Our contribution would allow universities to address the barriers to a successful graduate experience for both mobile and non-mobile students. Ultimately, interventions should be designed, evaluated, and the most promising implemented on a large scale. There is a growing need to explore the implications and to better understand the effects of mobility on postgraduates in various sociocultural and pedagogical situations. The aims of this study were to analyze the associations between LS and health-related factors, career attitudes and socioeconomic characteristics, and to determine factors that contribute to LS among mobile intra EU, mobile outside EU and non-mobile postgraduates who study in Luxembourg.

## Methods

### Population and design

A cross-sectional survey was conducted among the postgraduates who received financial aid from the government of Luxembourg, regardless of their socioeconomic situation. Registered in the database for the *Centre de Documentation et d’Information sur l’Enseignement Supérieur (CEDIES)*, 644 postgraduates were invited to participate in this survey, organized independently of the university.

### Procedure and ethical consideration

Students were contacted via an information flyer that was sent to their home addresses, inviting them to participate in a web survey. The flyer contained instructions about the goals of the study and the link to the self-administered survey. Consent was obtained from the respondents before the survey.

### Instruments and their translation

Participants could directly access the anonymous online questionnaire in either French or English. This had been translated and back-translated, and proofread by native-speaking professional translators.

### Data collection

#### Three groups of variables were collected:

##### * Life Satisfaction (LS)

Dependent variable, (one item, *how would you rate your life satisfaction?* from 1 very dissatisfied, to 10 very satisfied), partially verbal labels (endpoints). This metric does not include a neutral midpoint answering option [30, 31]. Satisfaction with one’s own life has been the focus of many cross sectional and longitudinal studies in the past decade, both within and across countries (OECD and Eurofound reports) [30, 31]. The most commonly used measures of life, evaluate ‘life as a whole’ or other similar over-arching constructs such as ‘Overall LS’ [31–33]. In addition to global judgments of life as a whole, it is also possible for people to provide evaluations of particular aspects of their lives (such as health or work) [34]. A strong relationship between overall life evaluations and evaluations of particular aspects of life exists. A meta-analysis [35] demonstrated that the asymptote for multiple item measures was the same as for single-item measures.

#### Additional variables:

##### * Health-related factors

- **Health satisfaction** (one item, *Are you satisfied with your health?* ranging from 1 very dissatisfied to 5 very satisfied) [30].
- **Quality of life autonomy**, a domain of the WHOQOL-BREF scale [36], QoL- autonomy *(*four items, from 1 strongly disagree to 5 strongly agree *- I have the freedom to make my own decisions; I feel control over my future; People around me are respectful of my freedom; I am able to do things I’d like).*
- **Worry** was measured with the Penn State Worry Questionnaire [37] (three items from 1 not at all typical, to 5 very typical of me - *Many situations make me worry; Once I start worrying, I cannot stop; I worry all the time).*

##### * Career attitudes

Four dimensions exploring attitudes [38] scored from 1 strongly disagree to 5 strongly agree.

- **Career adaptability** *(*four items, i.e. *I am good at adapting to new work settings; I can adapt to change my career plans; I can overcome potential barriers that may exist in my work; I can adapt to change in the world of work)*;
- **Career optimism** *(*four items*,* i.e. *I get excited when I think about my career; I understand- my work-related interests; I am unsure of my future career success; I am eager to pursue my career dreams);*
- **Career knowledge** *(*two items*,* i.e. *I am good at understanding job market trends; It is easy to see future employment trends);*
- **Career planning** *(*three items*,* i.e. *I have been thinking a lot about the type of job that best fits me; I have a clear plan for my career in the forthcoming years; I have been thinking a lot about what I want to realize in my job during the forthcoming years).*

##### * Socioeconomic characteristics

Age, sex, type of household (living alone/not) and perceived financial situation (1 very bad to 6 very good).

### Statistical analysis

Three groups were compared: postgraduates studying only in Luxembourg, mobile intra EU and mobile outside EU. To explore the associations between LS and sociodemographic variables, health-related factors and career attitude factors, student’s t-tests and bivariate correlations were used separately for each group. We estimated a single multiple regression model (Ordinary Least Squares) on the entire sample. We introduced the independent variable in interaction with the group variable to estimate each effect within each group of students. Only significant variables (*p* < 0.05) for at least one group were selected as explanatory variables. All scores were rescaled from one to 10 in order to make the estimated coefficients comparable. The analyses were performed using SPSS 22.0 software.

## Results

Of the 644, 490 respondents had indicated their country of study, and were included in the survey. The sample consisted of three groups: mobile outside EU (*n* = 43), intra EU (*n* = 381) university and non-mobile (*n* = 66).

### LS indicators and socioeconomic characteristics (Table 1)

Postgraduates who moved outside the EU (were mostly men and 63.2% of this group did not live alone) reported the highest LS. Intra EU and non-mobile groups were in majority women (57.8 and 51.6% respectively), and 64.3% (intra EU) vs. 93.7% (non-mobile) were not living alone. The non-mobiles who were studying in Luxembourg were the oldest group. For the outside EU group, autonomy of life, career adaptability and optimism scores were higher than for the other two groups. In contrast, planning and knowledge career attitudes were highest for the non-mobile.

**Table 1:** Description of LS scores, socioeconomic, health-related factors and career attitudes, for each group

Life Satisfaction [1–8, 12, 17]					
		Mobile outside EU = 43	Mobile intra EU = 381	Non mobile = 66	
		% or Mean (SD)	% or Mean (SD)	% or Mean (SD)	p^1^
		8.5 (1.5)	7.8 (1.6)	7.9 (1.5)	0.043*
Age		27.4 (8.1)	26.6 (5.2)	31.0 (8.5)	< 0.001***
Sex	Male	63.2	42.2	48.4	0.038*
	Female	36.8	57.8	51.6	
Type of household	Not living alone	57.9	64.3	93.7	< 0.001***
Financial situation	[1–8, 12, 17]	7.4 (2.7)	6.7 (2.2)	6.8(2.3)	0.157
Health-related factors [1–8, 12, 17]	Health satisfaction	7.9 (2.0)	7.4 (2.2)	7.4 (2.3)	0.438
	QoL-autonomy	7.5 (1.3)	6.8 (1.6)	6.9 (1.6)	0.052
	Worry	3.8 (2.4)	4.1 (2.4)	4.4 (2.4)	0.486
Career attitudes [1–8, 12, 17]	Adaptability	8.1 (1.1)	7.5 (1.4)	7.8 (1.6)	0.012*
	Optimism	7.8 (1.4)	7.1 (1.6)	7.1 (1.5)	0.034*
	Knowledge	5.7 (2.1)	5.6 (2.3)	6.0 (2.4)	0.440
	Planning	6.4 (2.1)	6.5 (2.3)	6.6 (2.0)	0.772

^1^ Significant *p*-value: *p < 0.05; **p < 0.01; ***p < 0.001 are presented in bold

### The relationships between LS scores and other variables (Table 2)

For outside EU, only the financial situation and all health-related factors were linked to LS. For intra EU, their financial situation was positively related to LS. All health-related factors and career adaptability, optimism and planning were also positively linked to LS Only worry, was negatively associated (− 0.426). For the non-mobile, health satisfaction, and career optimism and planning were positively linked to LS.

**Table 2:** Relationships between LS and socioeconomic, health factors and career attitudes for each group

Life Satisfaction [1–8, 12, 17]							
		Mobile outside EU		Mobile intra EU		Non-Mobile	
		Mean (SE)^1^	p^2^	Mean (SE)^1^	p^2^	Mean (SE)^1^	p^2^
Sex	Male	8.50 (0.28)	0.888	7.77 (0.12)	0.467	8.19 (0.23)	0.255
	Female	8.43 (0.45)		7.90 (0.11)		7.76 (0.30)	
Type of household	Living alone	8.75 (0.17)	0.334	7.71 (0.15)	0.207	7.50 (0.29)	0.586
	Not living alone	8.27 (0.40)		7.93 (0.10)		7.93 (0.20)	
		Correlation coefficient^3^	p^2^	Correlation coefficient^3^	p^2^	Correlation coefficient^3^	p^2^
Age		− 0.238	0.188	0.252	0.337	0.188	− 0.238
Financial situation	[1–8, 12, 17]	0.366	0.024*	0.117	0.026*	0.223	0.084
Health factors [1–8, 12, 17]	Health satisfaction	0.609	< 0.001***	0.437	< 0.001***	0.439	< 0.001***
	QoL-Autonomy	0.334	0.038*	0.429	< 0.001***	0.238	0.054
	Worry	−0.316	0.050*	− 0.426	< 0.001***	− 0.140	0.261
Career attitudes [1–8, 12, 17]	Adaptability	0.157	0.340	0.321	< 0.001***	− 0.037	0.767
	Optimism	0.139	0.399	0.388	< 0.001***	0.418	< 0.001***
	Knowledge	−0.061	0.711	0.101	0.052	0.197	0.114
	Planning	0.105	0.524	0.152	0.003**	0.366	0.002**

^1^Std. Error; ^2^Significant p-value: *p < 0.05; **p < 0.01; ***p < 0.001 are presented in bold; ^3^Pearson’s correlation

### Associations between LS score and other variables, for each group (Table 3**)**

Multiple OLS regression was used to determine the associations between life satisfaction and other variables. For all groups, health satisfaction was positively linked with LS. This association was strongest for the outside EU group, for whom it was moreover the only determinant contributing to LS. Besides health satisfaction, for intra EU mobiles, autonomy and career adaptability were positively associated to the LS score (regression parameter estimates (b): 0.210 and 0.119, respectively), and negatively to worry (b: − 0.153). For the non-mobile, career optimism and planning were also linked to their LS (b: 0.400 and 0.212, respectively).

**Table 3:** Multiple regression to determine the associations between health-related factors, career attitudes and LS, for each group

Life satisfaction [1–8, 12, 17]						
		b^1^	SE^2^	L95^3^	U95^4^	p^5^
Mobile outside EU						
Financial situation	[1–8, 12, 17]	0.037	0.087	−0.133	0.208	0.669
Health-related factors	Health satisfaction	0.368	0.120	0.132	0.603	0.002**
	QoL-Autonomy	0.305	0.253	−0.192	0.802	0.228
	Worry	−0.159	0.098	−0.351	0.033	0.104
Career attitudes	Adaptability	−0.026	0.236	−0.489	0.437	0.912
	Optimism	0.008	0.193	−0.372	0.388	0.967
	Planning	0.064	0.134	−0.200	0.328	0.634
Mobile intra EU						
Financial situation	[1–8, 12, 17]	0.040	0.032	−0.022	0.102	0.209
Health-related factors	Health satisfaction	0.193	0.033	0.129	0.257	≤0.001***
	QoL-Autonomy	0.210	0.055	0.102	0.318	≤0.001***
	Worry	−0.153	0.031	−0.213	− 0.092	≤0.001***
Career attitudes	Adaptability	0.119	0.054	0.014	0.224	0.027*
	Optimism	0.084	0.057	−0.028	0.197	0.141
	Planning	−0.004	0.034	−0.070	0.062	0.906
Non-mobile						
Financial situation	[1–8, 12, 17]	0.100	0.079	−0.057	0.256	0.211
Health-related factors	Health satisfaction	0.193	0.074	0.046	0.339	0.010**
	QoL-Autonomy	−0.175	0.158	−0.487	0.136	0.269
	Worry	−0.002	0.075	−0.149	0.145	0.978
Career attitudes	Adaptability	−0.068	0.132	−0.328	0.192	0.607
	Optimism	0.400	0.147	0.112	0.689	0.007**
	Planning	0.212	0.095	0.026	0.398	0.026*

^1^b = Parameter estimate; ^2^SE = Standard error; ^3^L95 = Lower limit of the 95% confidence interval; ^4^U95 = Upper limit of the 95% confidence interval; ^5^p = Significance level of the t-test: *p < 0.05; **p < 0.01; ***p < 0.001 are presented in bold

## Discussion

Our main findings show that the farther the mobility, the higher the LS. For all groups, the better the health satisfaction was, the higher the LS; confirming that health is a relevant indicator of LS [11]. In addition, autonomy and worry factors as well as career attitudes, i.e. adaptability, career optimism (aspirations) and planning explain some associations with LS. Mobility and LS highlight the contrast between local students (who present lower LS but higher optimism and planning) and cosmopolitan students (who present higher adaptability and LS). Below, we discuss the situation for each group.

For mobile outside-EU postgraduates, health satisfaction is the only determinant contributing to LS. Hence, all they need is to keep up their health situation to enhance their LS, which is necessary for their subsequent professional steps. European reports have identified perceived health status as the most notable predictor of LS [31], as well as an important component of overall well-being [30]. This major finding concurs with the fact that LS is related to positive outcomes as people who are satisfied with their lives, report lower levels of distress, which is predictive of future psychological behaviors [39, 40]. Students in mobility capitalize on their competencies, knowledge and their sociocultural development [18]. These mobile students may embody traits that are desirable in the labor market, e.g. language skills or cultural familiarity: ‘an overseas educational experience is believed to indicate (in its bearer) fluency in the English language as well as less obvious qualities, such as confidence, sociability, cosmopolitanism and possession of valuable social capital’ [41]. During their study period in a foreign country, students have to change their living habits, adapt to the host country’s conditions, adjust their professional goals to the labour market trends and develop their career attitudes further, to find a job [23].

For mobile intra EU graduates, the better their health-related factors (less worry, autonomy of life and health satisfaction) and the higher their career adaptability, the better their LS. International students have to cope with the financial and socio-cultural needs and psychological autonomy related to adjustment to the host country [21]; it is necessary to understand how they manage to turn a seemingly difficult situation into satisfying adaptation [42, 43]. The experience abroad permits the acquisition of new skills and competences. Consequently, they may feel more confident in their abilities and professional prospects and believe they made the right career choice, which would guarantee an interesting job. Studying abroad has been shown to cultivate the development of a positive set of values (self-esteem, self-efficacy and feeling useful) and lead to the acquisition of attributes which are important once in the labour market [15, 44, 45]. In contrast, the reverse may also be true, namely that self-selection among the more apt/ambitious students leads them to choose to study abroad – which leads to better outcomes among this group. Today, postgraduates must be able to manage the transition from their home country to their new country and to cope with obstacles related to their academic curriculum and their student life (psychological difficulties, socioeconomic circumstances, various socio-cultural relationships and additional requirements), which have an impact on their LS.

For the non-mobile group who pursued their studies in Luxembourg, in addition to health satisfaction as mentioned previously, their career optimism and planning attitudes were also determinants of LS. Their profile consisted of mainly women, who were also the oldest of the three groups of postgraduates (average age of 31 years) and the most likely to be living with family and to be working. A previous study among Luxembourgish native postgraduates [46] observed that higher health satisfaction, psychological quality of life and career optimism, were associated with higher LS. One hypothesis is that they saw themselves as leading a reasonably balanced lifestyle and they felt able to cope with their university studies. For our study postgraduates, the association between LS and the career attitudes (optimism and planning) showed that they possess local resources and social competences. Optimistic attitudes as the strongest determinant may imply that they believed their background gave them confidence in their knowledge in potential work sectors and/or tangible options for career advancement. In other words, under certain circumstances, as with the local postgraduates in our study, the incentives to stay in the home country, Luxembourg, may be higher as it allows building or increasing local cultural capital for getting ahead in(to) the home labor market. The importance of career planning attitudes seems to confirm their strategic motivation and rational focus on employability in their decision against mobility. In sum, this group seems to be at a different point in life where they have perspectives and strategic plans that may collide with spending time abroad. Additionally, in the international, multicultural and multilingual context of Luxembourg, much of the ‘foreign cultural capital’ may be accrued in the home country [47].

Contextualizing our results, general LS was higher for the postgraduates who studied overseas (8.5/10) compared to those who were mobile intra EU (7.8/10) and those who were non-mobile (7.9/10). The mobile outside EU were mostly men and 2/5 did not live alone. The non-mobile group who studied in Luxembourg was on average older and the majority did not live alone. Several explanations could be given for their stay. They were unable to stay in their own countries; probably lived as part of a couple or in a family with or without children. This likely means that they were possibly taking care one or more family member/s or they worked part time. The same reasons could be proposed for the mobile intra EU because mobility abroad can be achieved in the countries bordering Luxembourg, as it is easy to commute daily to and from home. It is notable that for the postgraduates outside EU (25–34 years), LS was higher than the national LS indicator in Luxembourg (7.7/10 in 2013). However, in our study, the scale was scored from 0 to 10, which gave a value of 7.93/ 10 when scored to from 1 to 10. In the EU-28, the score was 7.3/10 in 2013 - scored from 0 to 10; and 7.53 when scored from 1 to 10, for the same age group [48]. Another result from our study revealed that a correlation between LS and financial situation exists only among the mobile postgraduates. This finding confirms previous research [21] which pointed out that satisfaction with one’s financial situation is an important factor in predicting subjective well-being and which affects their LS, though no such link was found among the non-mobile university students.

### Strengths and limitations of our study

The main strength of the present study was the recruitment of a population of students legally residing in Luxembourg, who could pursue their Master’s degrees all over the world, thanks to the financial aid from the Luxembourgish government. Further, within the mobility, two types of students were identified (a) those who chose to go and (b) those who had no choice but to go. In the first case, they decided to study abroad in pursuit of their own interests; in the second case, they had to move, given that Luxembourg’s small size and young university did not locally offer their desired academic programs or courses.

As the OLS model was estimated on the entire sample, a common residual variance was estimated, which made it possible to maintain a reasonable power of the tests, in comparison with the adjustment of a distinct regression model for each group separately. Nevertheless, the low number of observations in the mobile outside EU group and the non-mobile group remains a limit which decreased the precision of the parameters estimated for these two groups. In other words, small effect sizes were more difficult to detect with fewer observations, increasing the risk of falsely confirming the null hypotheses.

## Conclusions

Mobile postgraduates possess a superior cultural capital gained through their higher adaptability and autonomy, suggesting that Bourdieu’s concept of cultural capital is a part of the social inequities puzzle. Thus, more research on the effects of cultural capital on LS and health is necessary. Furthermore, we recommend that LS among mobile and local students be measured periodically in all universities. Enhancing postgraduate students’ LS through cultural accompaniment, provision of counseling and orientation programs needs to be proposed by the host universities, to reduce inequality gaps.

In terms of cultural capital’s utility in health promotion, this study suggests that, in addition, university policy makers need to address health inequities and how its different elements may influence LS. Health promotion needs to promote an empowerment approach with a focus on student community mobilization that complements social initiatives. The challenge faced by universities, is to design strategies that will help students, especially the locals, to develop a ‘portfolio of resources’, linking them to a wealth of resources.

## Data Availability

Data and material are available at the Ethics Committee of the Institute for Research on Socio-Economic Inequalities, axis Health Inequalities.

## Abbreviations

CEDIES: Centre de Documentation et d’Information sur l’Enseignement Supérieur
Intra EU: Students who pursue part of their studies (example, Erasmus semester) in a European country, different that their home country Luxembourg
LS: Life satisfaction
Outside EU: Students who study a part of their courses (example, Erasmus global exchange) out of the European countries
QoL: Quality of life
